# *miR-590-5p* mediates mitochondrial respiration, proliferation, and apoptosis in thyroid carcinoma cells *via* fibroblast growth factor receptor substrate 2

**DOI:** 10.20945/2359-4292-2024-0410

**Published:** 2025-08-18

**Authors:** Penghui Wang, Xiaoli Hou, Wei Sun, Jiajie Chen, Yasen Cao, Hong Cheng

**Affiliations:** 1 Yangzhou University Medical College, Yangzhou, China; 2 Department of Clinical Laboratory, Affiliated Hospital of Yangzhou University, Yangzhou, China; 3 Department of Medical Science, Yangzhou Polytechnic College, Yangzhou, China; 4 Yangzhou University Medical College, Jiangsu Key Laboratory of Experimental & Translational Non-coding RNA Research, Institute of Translational Medicine, Yangzhou University, Yangzhou, China

**Keywords:** Thyroid carcinoma, miR-590-5p, FRS2, mitochondrial respiration, cell proliferation

## Abstract

**Objective:**

Thyroid carcinoma (TC) is the most common cancer of the endocrine system. Dysregulation of *microRNA-590-5p (miR-590-5p)* has been associated with various malignancies. Targeting mitochondrial respiration is beneficial in treating TC. This study aims to evaluate the role of *miR-590-5p* in the proliferation and apoptosis of TC cells *via* mediating mitochondrial respiration.

**Materials and methods:**

Reverse transcription quantitative polymerase chain reaction (qRT-PCR) was used to analyze differential expression of *miR-590-5p* in TC and para-cancerous tissues, normal thyrocytes, and TC cell lines. TC cells were transfected with agomiRNA negative control (agomiR-NC) or agomiRNA-590-5p (agomiR-590-5p). Cell counting kit 8 (CCK-8) assays, JC-1 staining, reactive oxygen species (ROS) measurements, and flow cytometry were used to detect cell proliferation, mitochondrial membrane potential (MMP), ROS levels, and apoptosis, respectively. The targeting relationship between *miR-590-5p* and fibroblast growth factor receptor substrate 2 (*FRS2*) was verified using dual-luciferase reporter assay. The role of *miR-590-5p* in tumor growth was analyzed in mouse xenograft tumors.

**Results:**

*miR-590-5p* was expressed at low levels in TC tissues and cells relative to normal tissues. Overexpression of *miR-590-5p* reduced TC cell proliferation, enhanced apoptosis, and inhibited mitochondrial respiration. *miR-590-5p* suppressed *FRS2* transcription in TC cells. Overexpression of *FRS2* reversed the effects of *miR-590-5p* overexpression, limiting mitochondrial respiration and proliferation, and promoting apoptosis. *In vivo*, overexpression of *miR-590-5p* suppressed xenograft tumor growth in mice by reducing the transcription of *FRS2*.

**Conclusion:**

*miR-590-5p* was poorly expressed in TC. Overexpression of *miR-590-5p* limited TC cell proliferation and promoted apoptosis by reducing mitochondrial respiration via decreased transcription of *FRS2*.

## INTRODUCTION

Thyroid carcinoma (TC) is one of the most common carcinomas of the endocrine system, and its incidence is increasing (^1^). In 2022, the new World Health Organization Classification of Endocrine and Neuroendocrine Tumors, Fifth Edition (WHO 5th), included a systematic classification of TC according to the cell of origin and clinical risk (^2^). The principal TC categories are follicular cell-derived neoplasms (FDNs) and parafollicular cell (C cell)-derived carcinomas, with FDNs further divided into three classes, namely, benign, low-risk, and malignant (^3,4^). Surgical resection is the standard approach for treating most TCs. Patients with low-risk, well-differentiated TC can be treated with surgery alone, while those with high-risk features may require further thyrotropin suppression and radioiodine therapy (^5^). Despite the significant advances in molecular testing and the discovery of promising therapeutics, the overall mortality of TC has not been significantly reduced in the past few years (^6,7^).

Mitochondria are critical organelles involved in energy production and molecular synthesis to maintain cellular activity (^8^). Mitochondrial dysfunction has been linked to the progression of various cancers (^9^). Mitochondrial respiration is essential for the production of oxygen and nutrients, and is harnessed to fulfill the bioenergetic and biosynthetic requirements of tumorigenesis (^10^). A previous study has shown that TC cells are more reliant on mitochondrial function than normal thyroid cells, and inhibition of mitochondrial respiration can induce apoptosis in TC cells (^11^). Reactive oxygen species (ROS) are small oxygen-derived molecules, and their overproduction results induces oxidative stress and the collapse of mitochondrial respiration, thereby contributing to TC cell apoptosis (^12^). Targeting mitochondrial respiration may thus offer a potential therapeutic strategy for TC.

MicroRNAs (miRNAs) are a class of small, non-coding, single-stranded RNAs that promote mRNA degradation or inhibit translation by binding to complementary sites in the 3′ untranslated regions of target mRNAs (^13^). miRNAs have been shown to be involved in a wide array of cellular processes, including proliferation, apoptosis, metastasis, and differentiation, and can function as potential biomarkers for early cancer detection, prognostic indicators, and therapeutic targets in TC (^14,15^). An in-depth characterization of the miRNA transcriptome in normal thyroid cells and papillary TC tissues showed significant dysregulation of 89 miRNAs in the TC tissues relative to normal thyroid tissues (^16^). One such miRNA, *miR-590*, has been reported to inhibit TC progression and mitigate mitochondrial dysfunction (^17,18^). Nevertheless, whether *miR-590-5p* regulates the proliferation and apoptosis of TC cells *via* modulation of mitochondrial respiratory function remains unknown.

Bioinformatic analysis has predicted a targeting relationship between *miR-590-5p* and fibroblast growth factor receptor substrate 2 (*FRS2*). The fibroblast growth factor (FGF) pathway is closely involved in cancer development and progression (^19^). All FGFs require the FGF receptor substrate (FRS) to initiate downstream signaling (^20,21^). FRS2 interacts with FGF receptors (FGFRs) *via* its phosphotyrosine-binding domain, and increased expression or activation of FRS2 has been linked to the onset of various cancers (^22^). FRS2 has been demonstrated to play a role in thyroid carcinogenesis triggered by TRK oncogenes (^23^). Specifically, FGFR signaling can protect mitochondrial functioning by limiting the production of ROS (^24^). However, the role of FRS2 in mitochondrial respiration in TC cells has not been investigated.

To date, there have been no studies of the mechanism by which *miR-590-5p* regulates mitochondrial respiration. In this study, we aimed to investigate the function of *miR-590-5p* in mitochondrial respiration in TC cells and thereby provide a novel theoretical basis for the treatment of TC.

## METHODS AND MATERIALS

### Ethics statement

This study was approved by the ethics committee of Yangzhou University Medical College. All participants provided written informed consent. The animal experiments followed the Guidelines for the Use and Management of Laboratory Animals and were approved by the Laboratory Animal Ethics Committee of Yangzhou University Medical College. Adequate measures were taken to minimize the number of animals used and the pain and discomfort of the mice.

### Collection of clinical samples

TC and para-cancerous tissues (at least 5 cm away from tumor tissues) were collected from 35 patients with TC (confirmed by clinical diagnosis and histopathology) in Yangzhou University Medical College from April 2015 to June 2019. None of the patients had received radiotherapy or chemotherapy before surgery.

### Cell culture and treatment

TC cell lines (TPC-1, B-CPAP, MDA-T120, and SW579) and the normal thyroid epithelial cell line Nthyori3-1 were obtained from the ATCC (Manassas, VA, USA). Cells were cultured in RPMI 1640 medium containing 10% fetal bovine serum at 37 °C with 5% CO_2_. All cells were identified by Short Tandem Repeat (STR) and showed no mycoplasma infection.

AgomiRNA for *miR-590-5p* overexpression (agomiR-590-5p) and agomiRNA negative control (agomiR-NC), *miR-590-5p* mimics and mimics-NC, *FRS2* overexpression vector pcDNA3.1-FRS2 (pc-FRS2) and the empty control vector pcDNA3.1-NC were purchased from Biomics (Jiangsu, China). Following the manufacturers’ instructions, the vectors were transfected for 48 h into TC cells using Lipofectamine 2000 (Invitrogen, Waltham, MA, USA). The *FRS2*-overexpression vector contained the full-length coding sequence (CDS) of *FRS2* (1527 bp), which was cleaved by HindIII and EcoRI and connected to the pcDNA3.1 plasmid (prokaryotic resistance: Amp [ampicillin]; eukaryotic resistance: NeoR/KanR [neomycin resistance/kanamycin resistance]).

Rutin hydrate (RH) was purchased from MedChemExpress (Monmouth Junction, NJ, USA) and added to cells at a concentration of 50 µM (^25^). Subsequent testing was performed after 24 h.

### Quantitative reverse transcription polymerase chain reaction (qRT-PCR)

The total RNA contents of TC cells, normal thyroid epithelial cells, and tumor samples were extracted using TRIzol (Invitrogen). The primers were designed and synthesized by Takara (Beijing, China) and the sequences are shown in **Table 1**. RNAs were transcribed to cDNA using Rever Tra Ace Qpcr RT Master Mix kits (TOYOBO, Osaka, Japan), and fluorescent quantitative PCR was performed using SYBR^®^ Premix Ex TaqTM II kits, as directed. The conditions for PCR were as follows: pre-denaturation at 94 °C for 4 min, followed by 30 cycles of denaturation at 94 °C for 30 s each, annealing at 59 °C for and extension at 72 °C for 1 min, followed by an additional extension at 72 °C for 5 min. The internal reference genes were *U6* for measuring *miR-590-5p* expression and *GAPDH* for measuring that of FRS2, with relative expression calculated using the 2-^∆∆Ct^ method. Each experiment was repeated three times independently, and the average value was used.

**Table 1:** Primer sequences

Name of primer	Sequences (5’-3’)
*miR-590-5p*	F:5’-GAGCTTATTCATAAAAGT-3’
	R:5’-TCCACGACACGCACTGGATACGAC-3’
*U6*	F:5’-GTGCTCGCTTCGGCA GCACAT-3’
	R:5’-TACCTTGCGAAGTGCTTA AAC-3’
*FRS2*	F: 5’CTGTCCAGATAAAGACACTGTCC-3’
	R:5’-CACGTTTGCGGGTGTATAAAATC-3’
*GAPDH*	F:5’-CCTGTTCGACAGTCAGCCG-3’
	R:5’-CGACCAAATCCGTTGACTCC-3’

### Cell Counting Kit-8 (CCK-8) assay

CCK-8 assays were used to assess cell viability. Cells were seeded into 96-well plates at a density of 2 × 10^3^/100 µL. Viability was measured at 0, 24, 48, and 72 h, measuring three replicates at each time point. The blank control wells contained medium without cells. The plates were cultured at 37 °C with 5% CO2. At each time point, 10 µL of CCK-8 reagent (Beyotime, Shanghai, China) was added to each well, followed by incubation for 4 h. Absorbance at 450 nm was measured using a microplate reader. Each experiment was repeated three times independently.

### Measurement of oxygen consumption rate (OCR)

Forty-eight hours after cell transfection, the ATP synthase inhibitor oligomycin, the mitochondrial uncoupling agent carbonyl cyanogen 4-(trifluoromethoxy) phenylhydrazine (FCCP), and antimycin/rotenone were added in succession to B-CPAP and MDA-T120 cells. An extracellular flow analyzer Seahorse-XF (Seahorse Bioscience, Chicopee, MA, USA) was used to measure the OCR. Each experiment was repeated three times independently.

Basic respiration represents the rate of oxygen consumption required by cells to maintain basic metabolism without any additives or interference. The decrease in OCR after the addition of oligomycin represents oxygen consumption dependent on ATP production. FCCP is used to relieve proton gradients, forcing mitochondria to consume oxygen at the maximum rate (maximal respiration). After FCCP treatment, OCR does not increase significantly, indicating damage to the mitochondrial electron transport chain or a lack of sufficient substrate. Rotenone inhibits mitochondrial complex I (NADH dehydrogenase) and impedes the production of ATP, which can be used to evaluate the reserve respiratory capacity and maximum respiratory potential of the cell. Mitochondrial dysfunction was evaluated based on the OCR values.

### Measurement of mitochondrial membrane potential (MMP)

The MMP was measured by JC-1 staining. At high MMP levels, JC-1 aggregates within the mitochondria to form a polymer that emits red fluorescence (589/590 nm). At reduced MMP levels, JC-1 is present in a monomeric form that emits green fluorescence (514/529 nm). TC cells were seeded into 6-well plates at a density of 1×10^5^/well. Cells were stained with JC-1 (C2006, Beyotime), as directed, with incubation with the solution for 10 min followed by imaging and using confocal laser scanning microscopy (FV3000, Olympus, Tokyo, Japan). Each experiment was repeated three times independently.

### Measurement of reactive oxygen species (ROS)

ROS was measured using ROS assay kits. Differently transfected cells were seeded in 6-well plates at a density of 1×10^5^/well and grown for 24 h, after which the cells were collected, lysed, and incubated in 10 mM 2ʹ, 7ʹ-dichlorofluorescein diacetate (DCFDA) for 20 min at 37 °C in the dark. ROS fluorescence was evaluated under a fluorescence microscope. Each experiment was repeated three times independently.

### Apoptosis detection

Apoptosis was examined using Annexin V-FITC/propidium iodide (PI) Apoptosis Detection kits (Beyotime) as directed. Briefly, differently transfected cells (1 × 10^6^) were resuspended in 100 µL binding buffer, which was then mixed with 2 µL Annexin-V-FITC (20 µg/mL), placed on ice in the dark for 15 min, and then transferred to flow testing tubes with the addition of 300 µL of phosphate-buffered saline (PBS). One microliter of PI (50 µg/mL) was added to each tube, and the cells were analyzed by flow cytometry within 30 min. Each experiment was repeated three times independently.

### Dual-luciferase reporter assay

TargetScan (https://www.targetscan.org/), StarBase (http://starbase.sysu.edu.cn), and miRTarBase (http://mirtarbase.cuhk.edu.cn) were used to predict the potential target genes of *miR-590-5p*. Then, dual-luciferase reporter assays were performed to verify the database predictions. After PCR amplification, the 3’UTR sequence of *FRS2* containing the *miR-590-5p* binding site was cloned into the downstream 3’ end of the PmiRglo dual luciferase target expression vector (Promega, Madison, WI, USA) to construct the *FRS2*-wild type (WT) reporter vector. Similarly, the *FRS2* mutant (MUT) 3ʹ-UTR sequence was designed to produce FRS2-MUT. FRS2-WT/MUT and miR-590-5p mimic/mimic-NC (Biomics, Jiangsu, China) were co-transfected into 293T cells in 12-well plates using Lipofectamine 2000. Each well contained 5 × 10^5^ cells together with 50 ng plasmid, with mimic concentrations of 20 nM. Luciferase activity was measured after 24 h on a fluorescence detector (Promega). Each experiment was repeated three times independently.

### Western blotting

Total protein was extracted from cells or tissues using RIPA buffer, and protein concentrations were determined using BCA assays. The protein samples were separated on SDS-PAGE and transferred to PVDF membranes (Millipore, USA). The membranes were blocked with 5% skim milk in TBST buffer for 1 h, and then incubated with primary antibodies against FRS2 (1:5000, ab183492, Abcam, UK) and β-actin (1:2000, ab8227, Abcam) overnight at 4°C. On the second day, the membrane was washed three times with TBST and incubated with secondary antibody IgG (1:5000, ab6721, Abcam) for 1 h. Finally, the protein bands were visualized using an enhanced chemiluminescence system (Millipore).

### Xenograft tumor formation

Nude mice (N = 24) aged 6 weeks were purchased from Beijing Vital River Laboratory Animal Technology Co., Ltd., Shanghai Branch (Shanghai, China, License No: SYXK (Shanghai) 2017-0014). Mice were randomly divided into two groups with 12 mice per group. Each mouse was injected subcutaneously into the ipsilateral axilla with 1 × 10^7^ B-CPAP cells (treated with agomiR-NC or agomiR-590-5p) in 200 µL. Tumor volumes were measured with vernier calipers every seven days. On Day 35, the mice were euthanized by intraperitoneal injection of pentobarbital sodium (dose > 200 mg/kg). The tumors were harvested and weighed. The tumor volume was calculated using the formula: V = 0.5×D×d_2_, where V is the volume; D is the longitudinal diameter; and d is the transverse diameter. The tumors from six randomly selected mice from each group were embedded in paraffin for the detection of Ki67. The remaining six tumors were homogenized and used to detect RNA and protein expression.

### Immunohistochemistry (IHC) staining

Tumor tissues were fixed with 4% paraformaldehyde and embedded in paraffin. After dewaxing and rehydration, the tissue was incubated with 3% H_2_O_2_ for 20 min to block endogenous peroxidase activity. Subsequently, the tissue sections were blocked with 10% fetal bovine serum at room temperature for 1 h, incubated overnight at 4 °C with an anti-KI67 antibody (ab15580, Abcam), and then incubated at room temperature with an anti-IgG antibody (ab6721, Abcam) for 30 min. The slides were treated with hematoxylin and the cell nuclei were counterstained. The sections were then dehydrated, sealed with neutral gel, and observed under a microscope (Olympus CKX51).

### Statistical analysis

All experiments were performed at least three times. FlowJo 10 (FlowJo, LLC, Ashland, OR, USA) was used to analyze the flow cytometry data. GraphPad Prism 8 (GraphPad Software, La Jolla, CA, USA) or SPSS 21.0 (IBM Corp, Armonk, NY, USA) was used for statistical analysis. Data with normal distribution, as shown by Kolmogorov-Smirnov tests, are presented as mean ± standard deviation (SD), and data between two groups were analyzed using *t-*tests. Comparisons among multiple groups were analyzed using one-way analysis of variance (ANOVA) or two-way ANOVA and checked by Tukey’s multiple comparisons test. *P-*values were obtained from two-tailed tests, and *P* < 0.05 was considered statistically significant.

## RESULTS

### *miR-590-5p* shows low expression in TC

**Figure 1:**
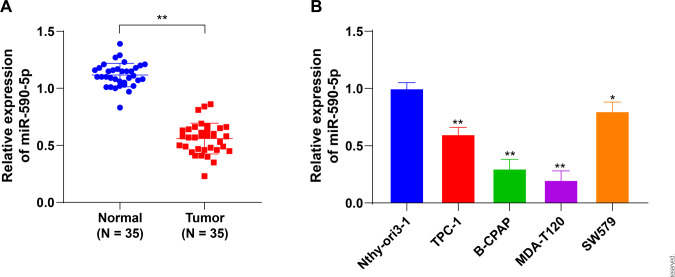
*miR-590-5p* shows low expression in TC. A: qRT-PCR measurement of *miR-590-5p* expression in TC tissues (N = 35); B: qRT-PCR measurement of *miR-590-5p* expression in TPC-1, B-CPAP, MDA-T120, SW579, and Nthyori3-1 cells. The relative expression of *miR-590-5p* was calculated by the 2^-∆∆Ct^ method using U6 as the internal reference, and normalized using expression in normal cells or Nthy-ori 3.1 normal epithelial cells. Each cell experiment was repeated three times, * *P* < 0.05, ** *P* < 0.01 vs. Normal or Nthy-ori 3.1 cells. Data in panel A were analyzed by t-tests, and data in panel B were analyzed by one-way ANOVA, followed by Tukey’s multiple comparisons test.

Previous studies have reported low levels of *miR-590-5p* in various cancers (^26^-^28^). However, the expression of *miR-590-5p* in TC is not known. We evaluated the expression of *miR-590-5p* in TC tissues (n = 35) and TC cell lines (TPC-1, B-CPAP, MDA-T120, SW579) using qRT-PCR, and found that miR-590-5p expression was low in both TC tissues and cell lines (*P* < 0.01, **Figure 1A-B**). Specifically, the lowest expression was seen in B-CPAP and MDA-T120 cells. Therefore, B-CPAP and MDA-T120 cells were selected for subsequent experiments.

### Overexpression of *miR-590-5p* inhibits mitochondrial respiration and promotes apoptosis in TC cells

**Figure 2:**
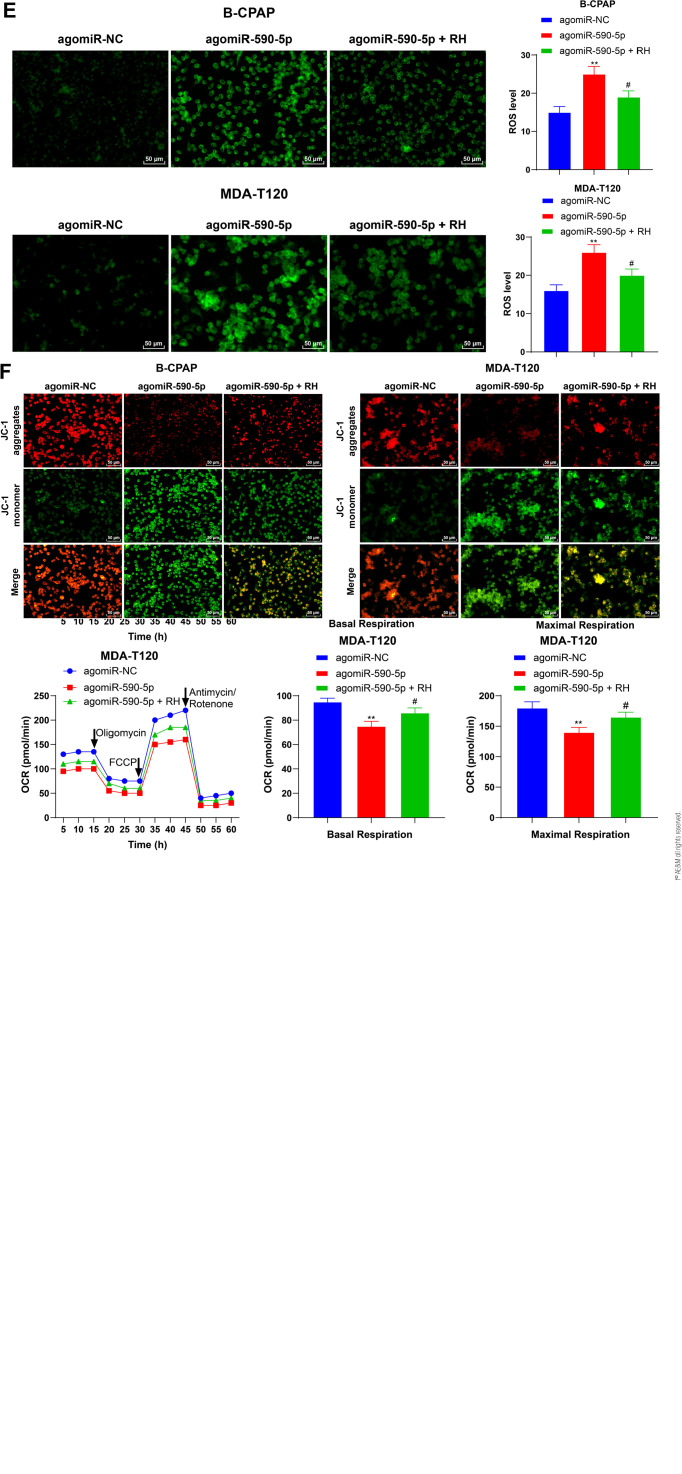
*miR-590-5p* overexpression inhibits proliferation and promotes apoptosis in B-CPAP and MDA-T120. agomiR-NC or agomiR590-5p was transfected into B-CPAP or MDA-T120 cells. The cells were divided into the agomiR-NC, agomiR590-5p, and agomiR590-5p + RH groups. **A:** qRT-PCR measurement of *miR-590-5p* expression. The relative expression of *miR-590-5p* was calculated by the 2^-∆∆Ct^ method, using U6 as the internal reference, and was normalized against agomiR-NC expression. **B:** CCK-8 assays showing cell viability; **C:** Flow cytometry examination of cell apoptosis; **D:** Seahorse XF measurements of OCR values of basal respiration and maximum respiration; **E:** Fluorescence labeling showing ROS levels; **F:** JC-1 staining to evaluate MMP. Each cell experiment was repeated three times. ** *P* < 0.01 vs. agomiR590-5p; # *P* < 0.05, ## *P* < 0.01. Data in panels ABC were analyzed by two-way ANOVA, followed by Tukey’s multiple comparisons test. Data in panels DE were analyzed by *t*-tests. OCR, oxygen consumption rate; RH, Rutin hydrate; agomiR-NC, agomiRNA negative control.

To further explore the role of *miR-590-5p* in TC cells, agomiR-590-5p was transfected into B-CPAP and MDA-T120 cells to overexpress *miR-590-5p* (*P* < 0.01, **Figure 2A**). After overexpression of *miR-590-5p*, TC cell viability was significantly reduced (*P* < 0.01, **Figure 2B**), while the apoptosis rate was increased (*P* < 0.01, **Figure 2C**). The OCR of cells treated with agomiR-590-5p was lower than that of agomiR-NC-treated cells after the addition of oligomycin and FCCP (*P* < 0.01, **Figure 2D**). Both basal and maximum OCR were significantly decreased (*P* < 0.01, **Figure 2D**), and ROS levels were increased (*P* < 0.01, **Figure 2E**). Moreover, the presence of green JC-1 fluorescence indicated a decrease in MMP (*P* < 0.01, **Figure 2F**). It was hypothesized that *miR-590-5p* regulates proliferation and apoptosis in TC cells by mediating mitochondrial respiration. Hence, agomiR-590-5p-treated TC cells were treated with the protectant of mitochondrial function RH (*P* < 0.01, **Figure 2B-F**). Compared with the agomiR590-5p group, the viability of TC cells was partly restored after the addition of RH (*P* < 0.01, **Figure 2B**), and the apoptosis rate was decreased (*P* < 0.01; **Figure 2C**). The results indicated that overexpression of *miR-590-5p* inhibited proliferation and promoted apoptosis in TC cells by reducing mitochondrial respiration.

### *miR-590-5p* inhibits *FRS2* transcription in TC cells

**Figure 3:**
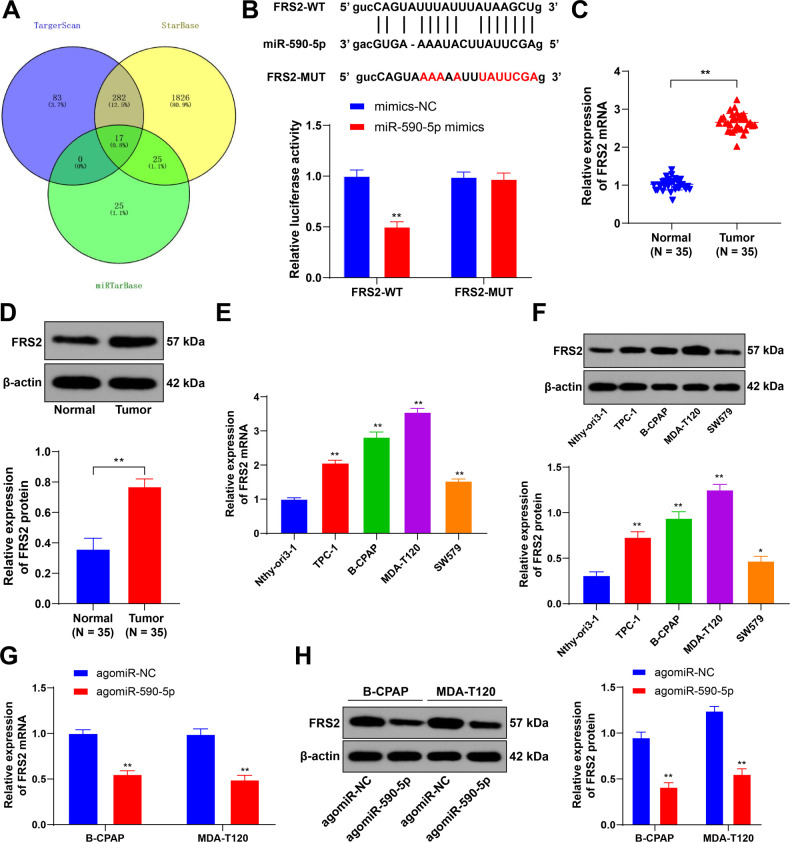
*miR-590-5p* inhibits *FRS2* expression in TC cells. **A:** TargetScan (https://www.targetscan.org/), StarBase (http://starbase.sysu.edu.cn) and miRTarBase (http://mirtarbase.cuhk.edu.cn) were used to predict downstream target genes of *miR-590-5p*, and predicted genes shared by the three databases were identified; **B:** Dual-luciferase reporter assays confirmed the targeting relationship between *miR-590-5p* and *FRS2*; **C-F:** qRT-PCR and Western blotting were used to assess *FRS2* expression in TC tissues (N = 35), para-cancerous tissues (N = 35), and TC cell lines (N = 3); **G-H:** qRT-PCR and Western blotting were used to assess *FRS2* expression in B-CPAP and MDA-T120 cells treated with agomiR-590-5p. N = 3. The relative expression of *FRS2* was calculated by the 2^-∆∆Ct^ method, with *GAPDH* as the internal reference, and normalized against Normal or agomiR-NC expression. * P < 0.05, ** P < 0.01 vs. mimics-NC in panel B, vs. Normal in panels CD, vs. Nthy-ori 3.1 cells in panels EF, vs. agomiR-NC in panels GH. Data in panels CD were analyzed by t-tests. Data in panels BGH were analyzed by two-way ANOVA, and data in panels EF were analyzed by one-way ANOVA, followed by Tukey's multiple comparisons test.

To explore the molecular role of *miR-590-5p* in TC cells, databases were used to predict the downstream target genes of *miR-590-5p*, and targets predicted by all three databases were identified (**Figure 3A**) with a specific focus on *FRS2*. Previous research has shown that *FRS2* expression is abnormal in papillary TC cells (^23^), and that *FRS2* promotes the growth of various tumors (^29^-^31^). Dual-luciferase reporter assays confirmed the targeting relationship between *miR-590-5p* and *FRS2*. Co-transfection of *miR-590-5p* mimics with FRS2-WT significantly reduced luciferase activity, while co-transfection with FRS2-MUT had no effect on luciferase activity (*P* < 0.01, **Figure 3B**). qRT-PCR measurements showed that the *FRS2* was highly expressed in TC tissues and cell lines (*P* < 0.05, **Figure 3C-F**). Furthermore, both mRNA and protein levels of *FRS2* were decreased after overexpression of *miR-590-5p* (*P* < 0.01, **Figure 3G-H**). Together, the results indicate that *miR-590-5p* inhibited *FRS2* transcription and protein expression in TC cells.

### Overexpression of *FRS2* reverses the effect of *miR-590-5p* overexpression on limiting mitochondrial respiration in TC cells

**Figure 4:**
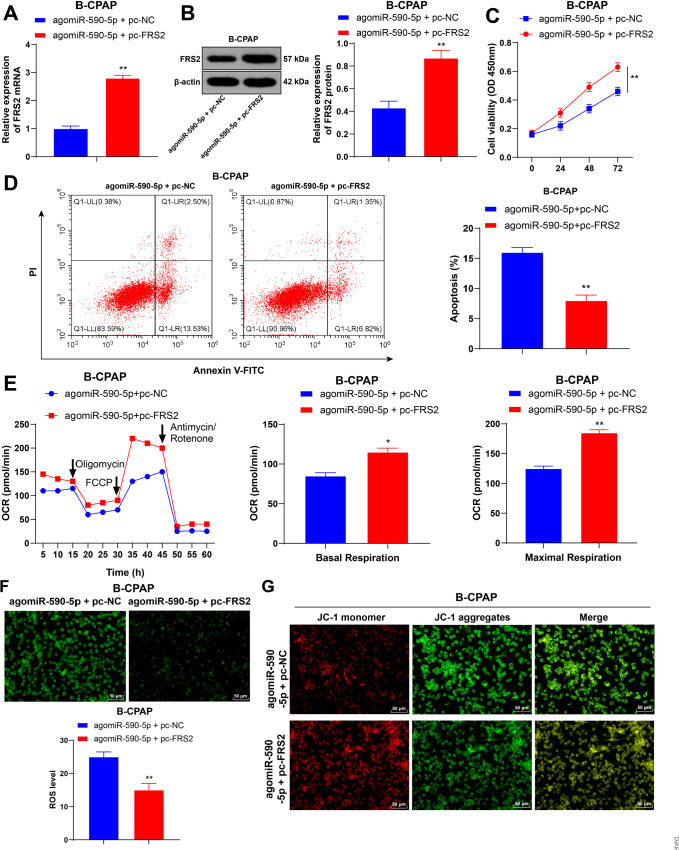
Overexpression of *FRS2* reduces the effects of *miR-590-5p* overexpression in TC cells. pc-*FRS2* or its blank control pc-NC was transfected into B-CPAP cells treated with agomiR-590-5p. **A-B:** qRT-PCR and Western blotting assessment of *FRS2* expression; **C:** CCK-8 assays were used to assess cell viability; **D:** Flow cytometry measurements of cell apoptosis; **E:** Seahorse XF was used to measure the OCRs of basal and maximum respiration; **F:** Fluorescence labeling of ROS levels; **G:** JC-1 staining to measure MMP. Each cell experiment was repeated three times. * *P* < 0.05, ** *P* < 0.01 vs. agomiR-590-5p + pc-NC. Data in panel C were analyzed by two-way ANOVA, followed by Tukey’s multiple comparisons test, and data in panels ABDEF were analyzed by *t*-tests. OCR, oxygen consumption rate; MMP, mitochondrial membrane potential.

To analyze the role of *FRS2* in TC cells, *FRS2* was overexpressed using *pc-FRS2* in agomiR-590-5p-treated B-CPAP cells (*P* < 0.01, **Figure 4A-B**). It was observed that overexpression of *FRS2* enhanced TC cell viability (*P* < 0.01, **Figure 4C**), decreased apoptosis rates (*P* < 0.01, **Figure 4D**), increased OCR values at all stages (*P* < 0.05, **Figure 4E**), reduced ROS levels (*P* < 0.01, **Figure 4F**), and increased the MMP (*P* < 0.01, **Figure 4G**). The results demonstrate that overexpression of *FRS2* could reverse the effects of *miR-590-5p* overexpression on limiting mitochondrial respiration and proliferation of TC cells while promoting apoptosis.

### Overexpression of *miR-590-5p* reduces xenograft tumor growth

**Figure 5:**
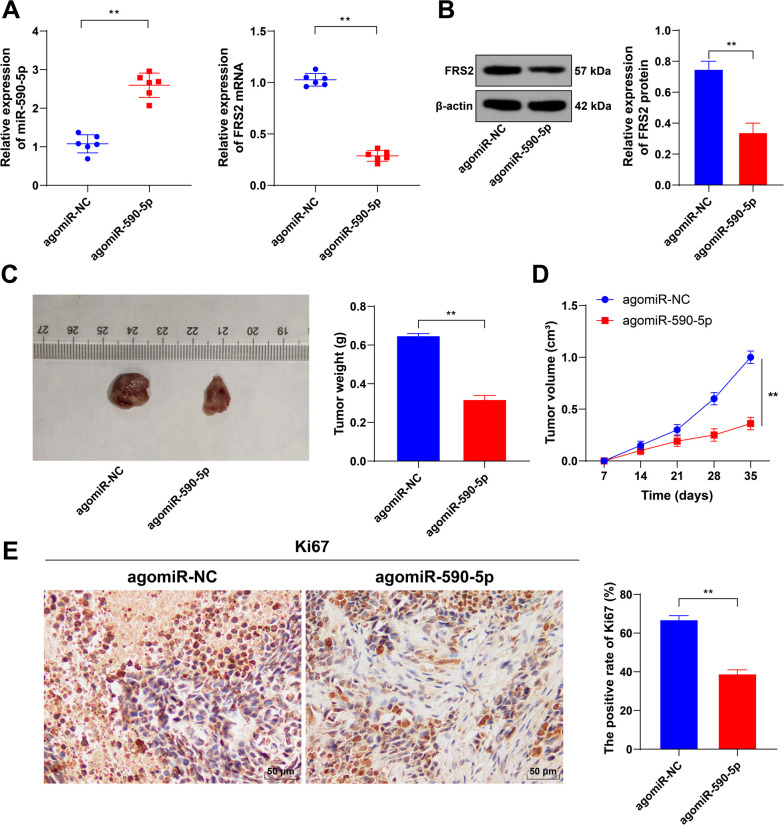
Overexpression of *miR-590-5p* slows TC growth *in vivo*. **A:** qRT-PCR measurement of *miR-590-5p* and *FRS2* expression; **B:** Western blotting evaluation of *FRS2* expression; **C-D:** Changes in tumor volumes and weights; E: KI67 immunohistochemical staining. N = 6. Data are presented as mean ± SD. Data in panel D were analyzed by two-way ANOVA, followed by Tukey's multiple comparisons test, and data in panels ABCE were analyzed by t-tests. ** *P* < 0.01 vs. agomiR-NC.

To verify the role of *miR-590-5p in vivo*, xenograft TC tumors were induced in mice, and the expression of *miR-590-5p* and *FRS2* in the tumor tissues was assessed. The results showed that after overexpression of *miR-590-5p* (*P* < 0.01, **Figure 5A**), the expression of both *FRS2* mRNA and protein was decreased (*P* < 0.01, **Figure 5A-B**), while tumor volumes and weights were significantly reduced (*P* < 0.01, **Figure 5C-D**), and KI67 positivity was decreased (*P* < 0.01, **Figure 5E**). Collectively, upregulation of *miR-590-5p* can mitigate TC tumor growth *in vivo via* reduced *FRS2* expression.

## DISCUSSION

TC is a relatively common endocrine malignancy, and recent evidence has shown the involvement of the mitochondria in its etiology (^32^). A broad range of miRNAs is documented to be abnormally expressed in TC (^33^). In the present study, *miR-590-5p* was found to inhibit TC progression *via* regulation of *FRS2* and mitochondrial respiration.

Many studies have reported the involvement of *miR-590-5p* in crucial tumorigenic processes, such as proliferation, invasion, metastasis, and chemo/radioresistance (^34^-^36^). We measured the expression of *miR-590-5p* in tissues from 35 TC patients and 4 TC cell lines (TPC-1, B-CPAP, MDA-T120, SW579) using qRT-PCR, and found that *miR-590-5p* was poorly expressed in both TC tissues and cell lines. After overexpression of *miR-590-5p*, TC cell proliferation was reduced, and apoptosis was enhanced. Tumor formation, progression, and metastasis are markedly dependent on mitochondrial function, specifically, the regulation of mitochondria-mediated apoptosis, Ca^2+^ homeostasis, energy production, and ROS generation (^11,37,38^). Mitochondrial respiration was assessed using OCR as a proxy for mitochondrial function. MMP is an indicator of mitochondrial activity. Overexpression of *miR-590-5p* reduced both the OCR and MMP after the addition of oligomycin and FCCP while increasing the levels of ROS, indicating damage to mitochondrial function and reduced mitochondrial respiration. The mitochondrial protective agent RH was then incubated with agomiR590-5p-treated TC cells. The results showed the restoration of TC cell viability to some extent with reductions in the apoptosis rate, indicating that overexpression of *miR-590-5p* inhibited proliferation and promoted apoptosis in TC cells *via* limiting mitochondrial respiration. *In vivo* mouse experiments showed that overexpression of *miR-590-5p* reduced the volumes and weights of the tumors. These results further verified that *miR-590-5p* could exert inhibitory effects on mitochondrial respiration, thereby inhibiting proliferation and promoting apoptosis in TC cells.

The downstream targets of *miR-590-5p* were then predicted using databases. Dual-luciferase reporter assays verified the targeted relationship between *miR-590-5p* and *FRS2*. FRS2 acts as a key adaptor protein in the FGFR pathway (^39^). Compelling evidence has shown the importance of FGF signaling in the pathogenesis of diverse tumor types, and clinical reagents that specifically target the FGFs or FGFRs are being developed (^40^). Since FRS is positioned at a critical juncture between the FGFR and downstream signal transduction, it is a potentially attractive target to disrupt the mitogenic and tumourigenic effects of multiple FGFs (^40^). For instance, FGFR signaling inhibits angiogenesis and tumor growth in hepatocellular carcinoma, and FGFR4-induced phosphorylation prevents apoptosis in breast cancer (^20,41^). In TC, FRS2 is activated by tropomyosin receptor kinase oncoproteins and promotes TC progression (^23^). In this study, qRT-PCR showed that *FRS2* was highly expressed in TC cells. Overexpression of *miR-590-5p in vitro* and *in vivo* reduced *FRS2* expression, indicating a negative correlation between *miR-590-5p* and *FRS2*. We subsequently overexpressed *miR-590-5p* and *FRS2* together in TC cells, and observed that TC cell proliferation was improved and the apoptosis rate was reduced, with increased OCR and MMP but decreased levels of ROS. These results indicated that overexpression of *FRS2* reversed the inhibitory effects of *miR-590-5p* on mitochondrial respiration and TC progression. Consistently, *FRS2* knockdown has been reported to act as a repressor of protein kinase D1, leading to inhibition of prostate cancer progression (^22^). In TC, FGFR2 downregulation competes with FGFR1 to reduce activation of FRS2 and the mitogen-activated protein kinase pathway, ultimately impeding TC progression (^42^). Moreover, FGFR1 plays a positive role in mitochondrial biogenesis and reduced ROS production, thereby enhancing mitochondrial respiration (^24^). Together, *miR-590-5p* inhibited mitochondrial respiration and thereby slowed TC progression by reducing retarded FRS2 expression.

In summary, *miR-590-5p* was found to reduce mitochondrial respiratory functions to block TC cell proliferation and induce apoptosis by targeting FRS2 expression. These findings suggest potential targets for the clinical treatment of TC.

There are also limitations to this paper. First, we did not investigate the role of *miR-590-5p* in other cellular functions of TC cells apart from mitochondrial respiration, cell proliferation, and apoptosis. Second, the role of other downstream targets of *miR-590-5p* regulating mitochondrial respiration was not explored. In the future, we will investigate the role of *miR-590-5p* in other cellular functions in TC cells and examine the involvement of other target genes downstream of *miR-590-5p* mediating mitochondrial respiration to verify the present findings.
